# Decoding the anti-hypertensive mechanism of α-mangostin based on network pharmacology, molecular docking and experimental validation

**DOI:** 10.1186/s10020-024-01001-8

**Published:** 2024-11-26

**Authors:** Qi-Qi Xue, Chu-Hao Liu, Yan Li

**Affiliations:** Department of Cardiovascular Medicine, Shanghai Key Laboratory of Hypertension, Shanghai Institute of Hypertension, National Research Centre for Translational Medicine, Ruijin Hospital, Shanghai Jiaotong University School of Medicine, Ruijin 2nd Rd 197, Shanghai, 200025 China

## Abstract

**Background:**

Hypertension is a leading risk factor for disability and deaths worldwide. Evidence indicates that alpha-mangostin(α-MG) can reduce blood pressure and improve target organ damage. Nonetheless, its pharmacological targets and potential mechanisms of action remain inadequately elucidated.

**Method:**

We used SwissTargetPrediction to identify α-MG’s drug targets and DisGeNET, GeneCards, CTD, and GEO databases for hypertension-related targets, and then determined antihypertensive therapeutic targets of α-MG by intersecting these targets. GO functional enrichment analysis, KEGG pathway analysis, and disease association analysis were conducted using the DAVID database and R package “clusterprofile”, visualized with Cytoscape software. The binding affinity of α-MG to identified targets was confirmed through molecular docking using Autodock Vina v.1.2.2 software. The impact of α-MG on target genes was validated using an Angiotensin II-induced hypertensive mouse model and RT-qPCR.

**Results:**

A total of 51 potential antihypertensive therapeutic targets for α-MG were identified by intersecting 109 drug targets with 821 disease targets. Furthermore, 10 cellular component terms, 10 disease terms, and the top 20 enriched biological processes, molecular functions, and KEGG pathways related to α-MG’s antihypertensive effects were documented. Molecular docking studies indicated a strong binding affinity of α-MG with the HSP90AA1 domain. In Ang II-induced hypertensive mice aorta, treatment with α-MG effectively reversed the aberrant mRNA expression of TNF, HSP90AA1, NFKB1, PPARG, SIRT1, PTGS2, and RELA.

**Conclusion:**

Our analyses showed that TNF, HSP90AA1, NFKB1, PPARG, SIRT1, PTGS2, and RELA might be α-MG’s potential therapeutic targets for hypertension, laying groundwork for further investigation into its pharmacological mechanisms and clinical uses.

**Supplementary Information:**

The online version contains supplementary material available at 10.1186/s10020-024-01001-8.

## Background

Hypertension is a major public health challenge afflicting over 1.1 billion people worldwide (Kurniansyah et al. 2022; Roth et al. 2020; Zhang et al. 2021). Uncontrolled hypertension has a significant negative impact on cardiovascular morbidity and mortality (Kario et al. 2021). The pathogenesis of hypertension is the result of multifactorial and multisystemic effects, including genetic factors, imbalances in neurohumoral regulation, abnormal fluid volume retention, alterations in vascular structure and function, metabolic abnormalities, and environmental and lifestyle factors (YJ et al. 2017; Grassi et al. 2015; Siebenhofer et al. 2021). Strict management of blood pressure, as recommended in the guidelines for the treatment of hypertension, is essential for the prevention of cardiovascular and renal events (Aronow and Frishman 2018). Despite advances in pharmacotherapy over the past few decades, hypertension has still not been well controlled (Mancia et al. 2023). Current guidelines recommend five main drug classes for the treatment of hypertension: angiotensin converting enzyme (ACE) inhibitors, angiotensin receptor blockers (ARBs), β-blockers, calcium channel blockers (CCBs), and diuretics (Mancia et al. 2023). Patients receiving antihypertensive drugs may suffer from side effects such as ACE inhibitor-induced cough and diuretics-associated hyperuricemia (Ueno et al. 2016; Arendse et al. 2019). Thus, developing novel anti-hypertensive drugs is urgently needed.

Natural products, especially plant extracts, have been an excellent source of drug discovery. Various plant extracts have shown potential in antihypertensive therapy (Marunaka et al. 2017; Jaleel et al. 2006). Alpha-mangostin (α-MG) is a natural xanthone derived from the pericarp of the mangostan fruit (Garcinia mangostana Linn.) and exhibits potential therapeutic benefits against various diseases due to its anti-inflammatory and antioxidant properties (Bumrungpert et al. 2009; Valdés-Torres et al. 2022; Jiang et al. 2021; Dang et al. 2023). One previous study has indicated the anti-hypertensive effect of mangostan fruit extract in L-NAME-induced hypertensive rats via suppressing oxidative stress and increasing NO bioavailability (Boonprom et al. 2017). However, the underlying anti-hypertensive mechanism of α-MG remains to be elucidated.

Network pharmacology is an innovative approach that integrates biological network analysis to understand drug interactions and therapeutic mechanisms (Hopkins 2008). It leverages databases to identify potential drug targets and pathways in the context of various diseases (Kibble et al. 2015). By employing methods like molecular docking and experimental validation, network pharmacology enhances the understanding of the multifaceted interactions of herbal medicines and their active targets (Yao et al. 2024; Wu et al. 2024; Deng et al. 2024).

**Fig. 1:**
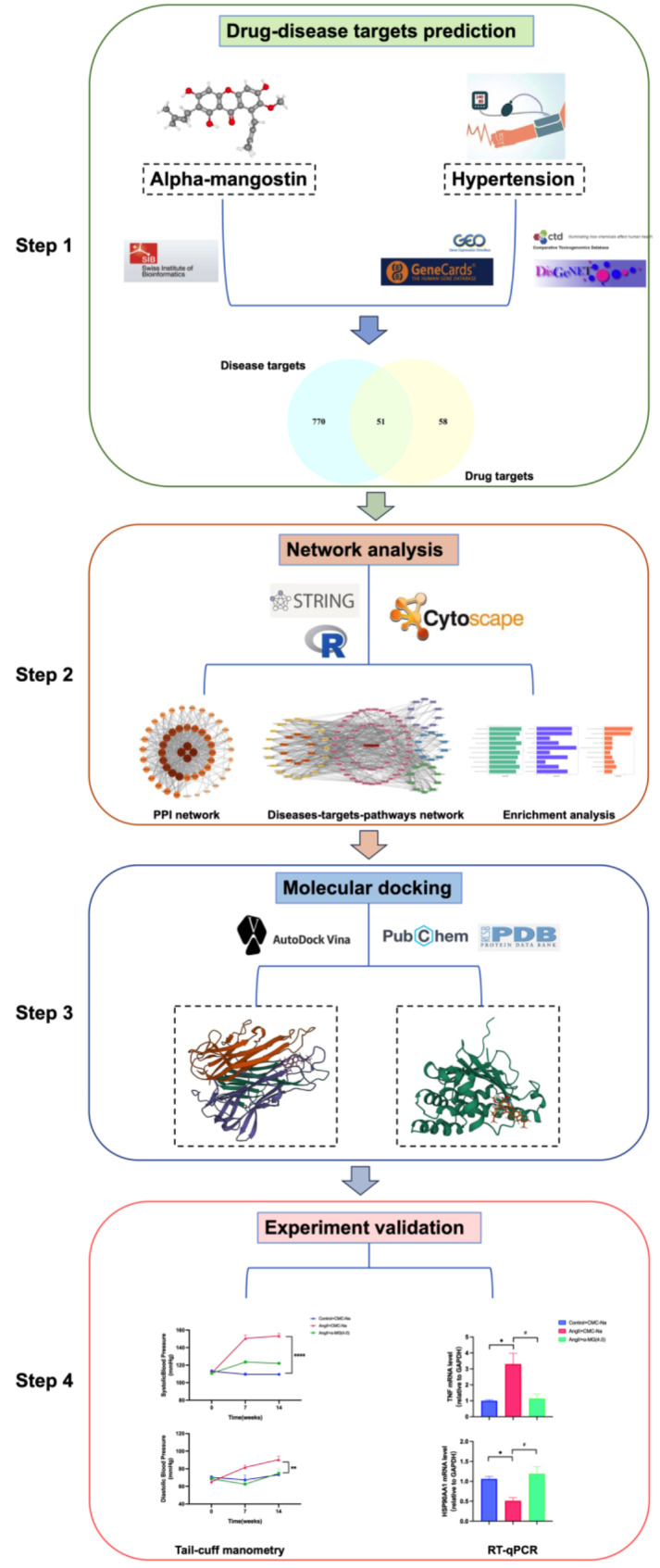
Flowchart of network pharmacology to identify the bioactive components and potential mechanisms of alpha-mangostin. PPI, protein-protein interaction

In this study, we aimed to decode the potential anti-hypertensive mechanisms α-MG through network pharmacology integrated with molecular docking and in vivo experimental validation. The flowchart is shown in Fig. 1.

## Materials and methods

### Drug targets of α-MG

SwissTargetPrediction provided the targets of α-MG (http://www.swisstargetprediction.ch). The SwissTargetPrediction database utilizes the similarity of two-dimensional and three-dimensional chemical structures of compounds to predict the most probable protein targets of small molecule drugs. It can enhance prediction accuracy based on prediction scores (Chen et al. 2023).

### Therapeutic targets of hypertension

Hypertension-related genes were retrieved from the DisGeNET database (http://www.disgenet.org/web/DisGeNET/menu/home), GeneCard database (https://www.genecards.org) and comparative toxicogenomics database (CTD database, http://ctdbase.org/). We collected genes associated with hypertension and essential hypertension. Microarray gene expression profiles of peripheral blood leukocytes were obtained from the GEO database (GSE74144, https://www.ncbi.nlm.nih.gov/geo/) based on the GPL13497 platform. The dataset included samples from 8 healthy individuals (Control, numbered GSM1911593-GSM1911600), 14 hypertensive patients without left ventricular hypertrophy (HT without LVH, numbered GSM1911565-GSM1911578), and 14 hypertensive patients with left ventricular hypertrophy (HT with LVH, numbered GSM1911579-GSM1911592). The R package “limma” was used to screen for differentially expressed genes (DEGs) between the control group and the HT without LVH group. After taking the concatenated set of all databases and genes obtained from differential analysis, we selected the genes with a frequency of 2 and above as hypertension-related target genes for subsequent analysis. Venn diagrams were drawn using the Venn package in R (4.0.2, May 2024).

### Construction of protein-protein interaction (PPI) network

Using the STRING database (https://string-db.org/), we constructed a protein-protein interaction (PPI) network based on therapeutic targets for α-MG against hypertension (Szklarczyk et al. 2021). We set the minimum required interaction score to “medium confidence” (0.400) to screen for interactions. The PPI enrichment p-value is set to < 1.0e-6. Following the construction of the PPI network, we analyzed the Degree (DC) values for each target using the CytoNCA plug-in for Cytoscape (Tang et al. 2015). The visualization of the generated PPI network was accomplished using Cytoscape software(3.10, May 2024) (Shannon et al. 2003).

### Construction of the network of diseases-targets‐functional annotations‐signaling pathways

We used the “clusterProfiler” R package to conduct gene ontology (GO) enrichment analysis, which includes GOTERM_BP_DIRECT, GOTERM_CC_DIRECT, and GOTERM_MF_DIRECT categories. Kyoto Encyclopedia of Genes and Genomes (KEGG) pathway analyses and disease association analyses were conducted using the DAVID database (accessed May 2024) (Dennis et al. 2003). The top 20 enriched KEGG pathways, top 10 GO-rich terms, and top 10 enriched disease associations with *P* < 0.05 were selected for further network construction. The disease-target-functional annotation-signaling pathway network was visualized using Cytoscape software.

### Molecular docking

To assess the binding affinity of α-MG to core targets, we performed molecular docking analysis. We obtained the interactions between α-MG and 20 target proteins using Autodock Vina v.1.2.2(May 2024).

### Experimental animals

In accordance with the Guide for the Care and Use of Laboratory Animals, the animal care and use committee of Shanghai Jiaotong University (China) approved the experimental protocols. SF-grade C57BL/6 mice aged 8 weeks were purchased from Shanghai JieSiJie Laboratory Animal Co., Ltd. The mice were kept at 25 ± 1 °C and 65 ± 5% humidity and acclimatized under a 12-hour light/dark cycle in the animal house of Ruijin Hospital, Shanghai Jiaotong University School of Medicine. Prior to experimentation, the mice were allowed to adapt to the environment for 1 week.

To induce hypertension, male C57BL/6 mice (8 weeks) were infused with Ang II (500 ng/kg/min) for 2 weeks through osmotic minipump subcutaneously implanted under isoflurane anesthesia. The model and the dose of Ang II were selected based on the AHA Scientific Statement (Lerman et al. 2019). Mice were randomly allocated into five distinct groups: a control group (*n* = 9), an Angiotensin II (Ang II) infusion group (*n* = 9), two groups receiving Ang II infusion along with gavage administration of α-MG at doses of 4.0 mg/kg (*n* = 9) and 8.0 mg/kg (*n* = 6), respectively, and a positive control group receiving Ang II infusion with gavage administration of Captopril at a dose of 50 mg/kg(*n* = 6). All treatments were administered via gavage, with the experimental period and conditions standardized across all groups. The control and Ang II-infused groups were gavaged with 0.5% sodium carboxymethylcellulose (CMC-Na), whereas the other groups received either α-MG or Captopril dissolved in 0.5% CMC-Na. All mice were administered at a volume of 0.2 ml twice a day at 9:00 and 17:00. Treatments were repeated at least in three independent groups of animals in different time periods.

### Tail-cuff blood pressure measurement

We used a non-invasive tail-cuff method with an animal sphygmomanometer (BP2010A, Softron Co., Ltd., Tokyo, Japan). Before the initial measurements, the mice and rats underwent a one-week training and acclimation period. Each blood pressure reading was determined by averaging a minimum of 3–5 stable consecutive measurements.

### Reverse transcription-quantitative polymerase chain reaction (RT‐qPCR)

Total RNA in mice aorta was extracted using RNA isolation kit (Promega). Real-time quantitative PCR was conducted with the QuantStudio 7 Flex system (Applied Biosystems) and the SYBR Green PCR Master Mix to measure the relative mRNA levels of the selected targets. These targets were chosen based on their degree values in the PPI and target-pathway networks. The primer sequences are shown in Table 1 and the detailed data of the mRNA levels are shown in Table S1.

**Table 1:** Primers of quantitative real-time PCR

Target	Sequence Forward Primer	Sequence Reverse Primer
TNF	CCCTCACACTCAGATCATCTTCT	GCTACGACGTGGGCTACAG
AKT1	ATGAACGACGTAGCCATTGTG	TTGTAGCCAATAAAGGTGCCAT
SRC	GAACCCGAGAGGGACCTTC	GAGGCAGTAGGCACCTTTTGT
CTNNB1	ATGGAGCCGGACAGAAAAGC	CTTGCCACTCAGGGAAGGA
HSP90AA1	AATTGCCCAGTTAATGTCCTTGA	CGTCCGATGAATTGGAGATGAG
NFKB1	ATGGCAGACGATGATCCCTAC	TGTTGACAGTGGTATTTCTGGTG
HSP90AB1	GTCCGCCGTGTGTTCATCAT	GCACTTCTTGACGATGTTCTTGC
PPARG	TCGCTGATGCACTGCCTATG	GAGAGGTCCACAGAGCTGATT
MTOR	ACCGGCACACATTTGAAGAAG	CTCGTTGAGGATCAGCAAGG
MAPK3	TCCGCCATGAGAATGTTATAGGC	GGTGGTGTTGATAAGCAGATTGG
CCND1	GCGTACCCTGACACCAATCTC	CTCCTCTTCGCACTTCTGCTC
SIRT1	GCTGACGACTTCGACGACG	TCGGTCAACAGGAGGTTGTCT
PTGS2	TGAGCAACTATTCCAAACCAGC	GCACGTAGTCTTCGATCACTATC
MAPK1	GGTTGTTCCCAAATGCTGACT	CAACTTCAATCCTCTTGTGAGGG
BCL2L1	GACAAGGAGATGCAGGTATTGG	TCCCGTAGAGATCCACAAAAGT
PIK3CA	CCACGACCATCTTCGGGTG	ACGGAGGCATTCTAAAGTCACTA
RELA	AGGCTTCTGGGCCTTATGTG	TGCTTCTCTCGCCAGGAATAC
HDAC1	AGTCTGTTACTACTACGACGGG	TGAGCAGCAAATTGTGAGTCAT
PRKCA	GTTTACCCGGCCAACGACT	GGGCGATGAATTTGTGGTCTT
APP	TCCGAGAGGTGTGCTCTGAA	CCACATCCGCCGTAAAAGAATG
GAPDH	AGGTCGGTGTGAACGGATTTG	TGTAGACCATGTAGTTGAGGTCA

### Statistical analysis

Continuous data are presented as the mean ± SD or mean ± SEM. Differences between multiple groups were evaluated using two-way analysis of variance (ANOVA) followed by Bonferroni post hoc tests. A *P*-value of less than 0.05 was considered statistically significant. All statistical analyses were conducted using GraphPad Prism 7.0.

## Results

### Identification of α-MG drug targets and disease targets

**Fig. 2:**
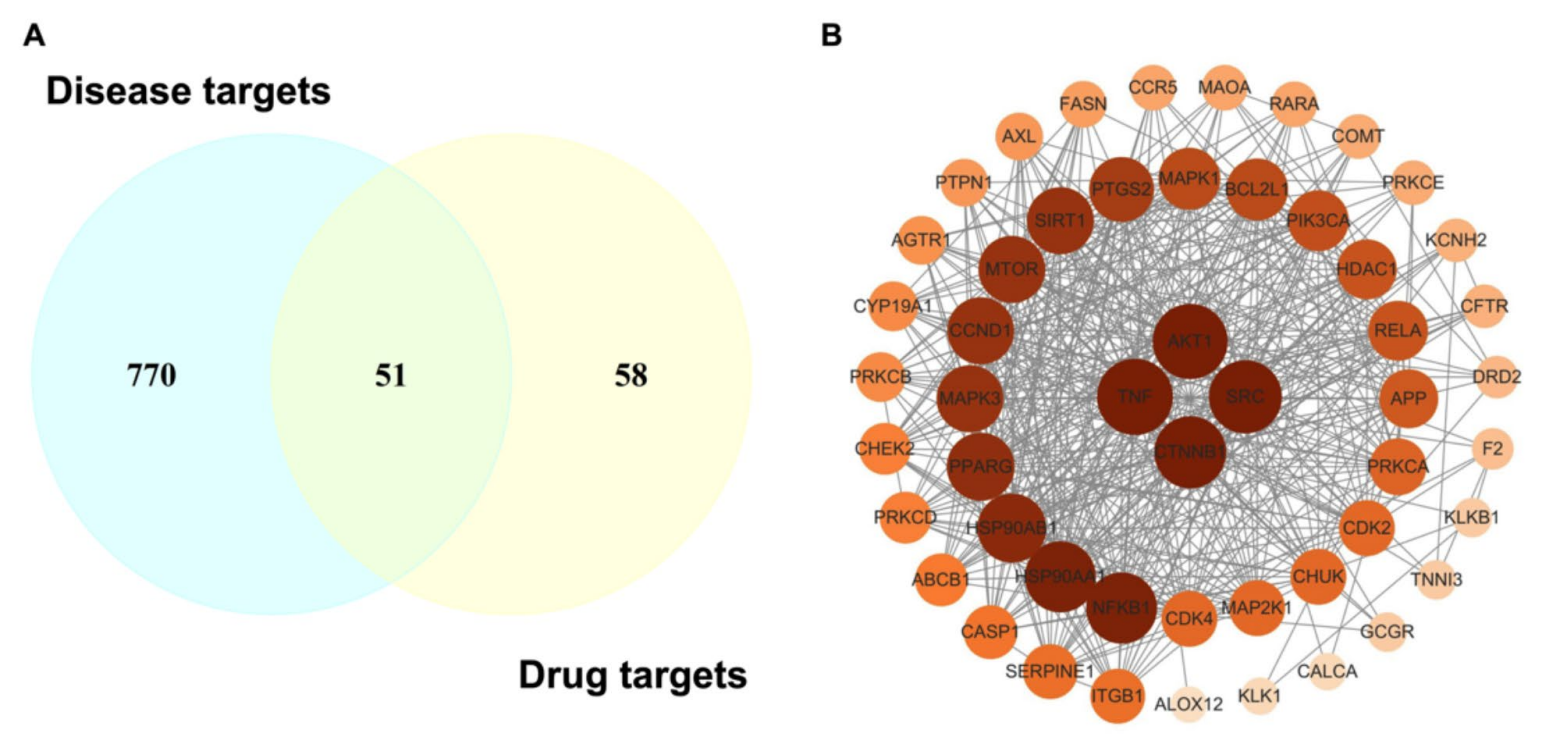
Identification of potential therapeutic targets of α-MG for hypertension. **A** Venn diagram to acquire the intersection of genes as potential therapeutic targets. **B** PPI network of α-MG targets against hypertension. The color of the nodes reflects the degree of connectivity (the redder color indicates a higher degree)

There were 109 α-MG drug targets identified using the SwissTargetPrediction database. To compile the disease targets for hypertension, we searched four databases: DisGeNET, GeneCards, CTD, and GEO databases. In GeneCards, a relevance score greater than 5 was used as the screening criterion (Tan et al. 2020). For DisGeNET, genes with a GDA score greater than 0 were selected (Chen et al. 2020). In the CTD, genes for the disease “hypertension” or “essential hypertension” were considered disease targets (Sun et al. 2023; Li et al. 2021; Zhan et al. 2023). GSE74144 was selected from the GEO database, and differentially expressed genes (DEGs) were identified using a threshold of |log FC| > 1 and a *P*-value < 0.05. This analysis yielded 25 DEGs out of 21,750 genes. After combining the results from all databases, we identified 821 genes with a frequency of 2 or more as hypertension-related targets. By intersecting these 821 disease-related genes with potential α-MG targets, we identified 51 overlapping targets. These overlapping targets were recognized as potential therapeutic targets for α-MG in the treatment of hypertension (Fig. 2).

### Construction of PPI network

The 51 identified therapeutic targets of α-MG against hypertension were used to construct a protein-protein interaction (PPI) network. This network consists of 51 nodes and 482 edges, with an average node degree of 18.9. Based on the degree value, the top 20 core targets include TNF, AKT1, SRC, CTNNB1, HSP90AA1, NFKB1, HSP90AB1, PPARG, MTOR, MAPK3, CCND1, SIRT1, PTGS2, MAPK1, BCL2L1, PIK3CA, RELA, HDAC1, APP, and PRKCA (Table 2).

**Table 2:** Top 20 targets of α-MG against hypertension ranked by degree value in the analyses of protein-protein interaction network

Gene name	Degree	Betweenness Centrality	Closeness Centrality
TNF	40	0.085511599	0.833333333
AKT1	39	0.063383225	0.819672131
SRC	37	0.066380403	0.793650794
CTNNB1	36	0.061787624	0.78125
HSP90AA1	35	0.035476483	0.769230769
NFKB1	35	0.029530591	0.769230769
HSP90AB1	33	0.026471994	0.746268657
PPARG	32	0.021790851	0.735294118
MTOR	31	0.013282423	0.724637681
MAPK3	31	0.015646553	0.724637681
CCND1	31	0.016101431	0.714285714
SIRT1	31	0.020328225	0.724637681
PTGS2	29	0.049793899	0.704225352
MAPK1	26	0.011812955	0.675675676
BCL2L1	26	0.004870156	0.666666667
PIK3CA	25	0.006625192	0.649350649
RELA	24	0.004263533	0.657894737
HDAC1	24	0.006113887	0.649350649
APP	23	0.055111271	0.649350649
PRKCA	21	0.007579514	0.625

### Diseases-targets‐functional annotations‐signaling pathways network

**Fig. 3:**
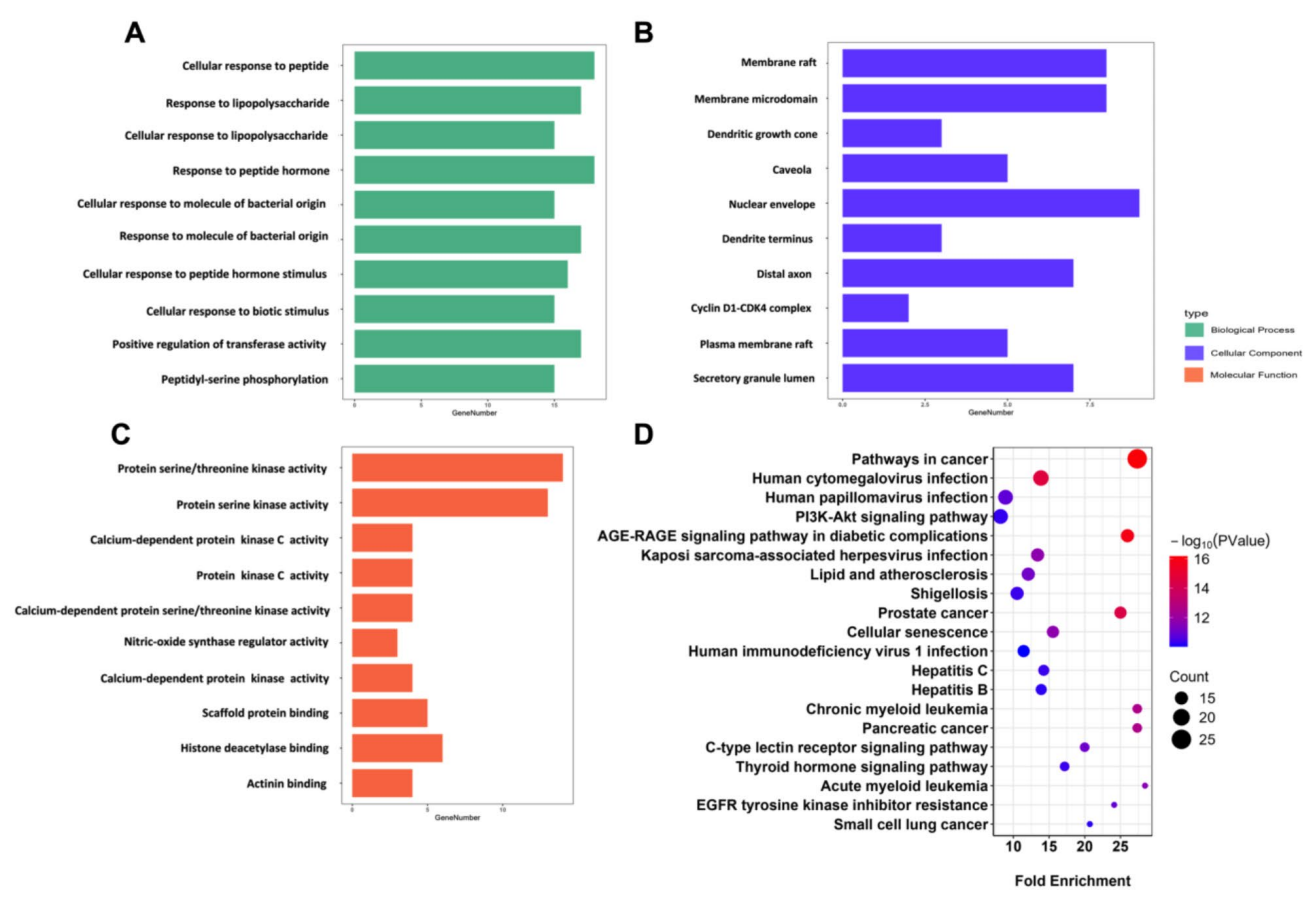
GO/KEGG analysis of the potential therapeutic targets. **A** Top 10 enriched GO terms of BP, CC, MF. **B** Top 20 enriched KEGG pathways. GO: Gene ontology; KEGG: Kyoto Encyclopedia of Genes Genomics; BP, biological process; CC, cellular component; MF, molecular function

**Fig. 4:**
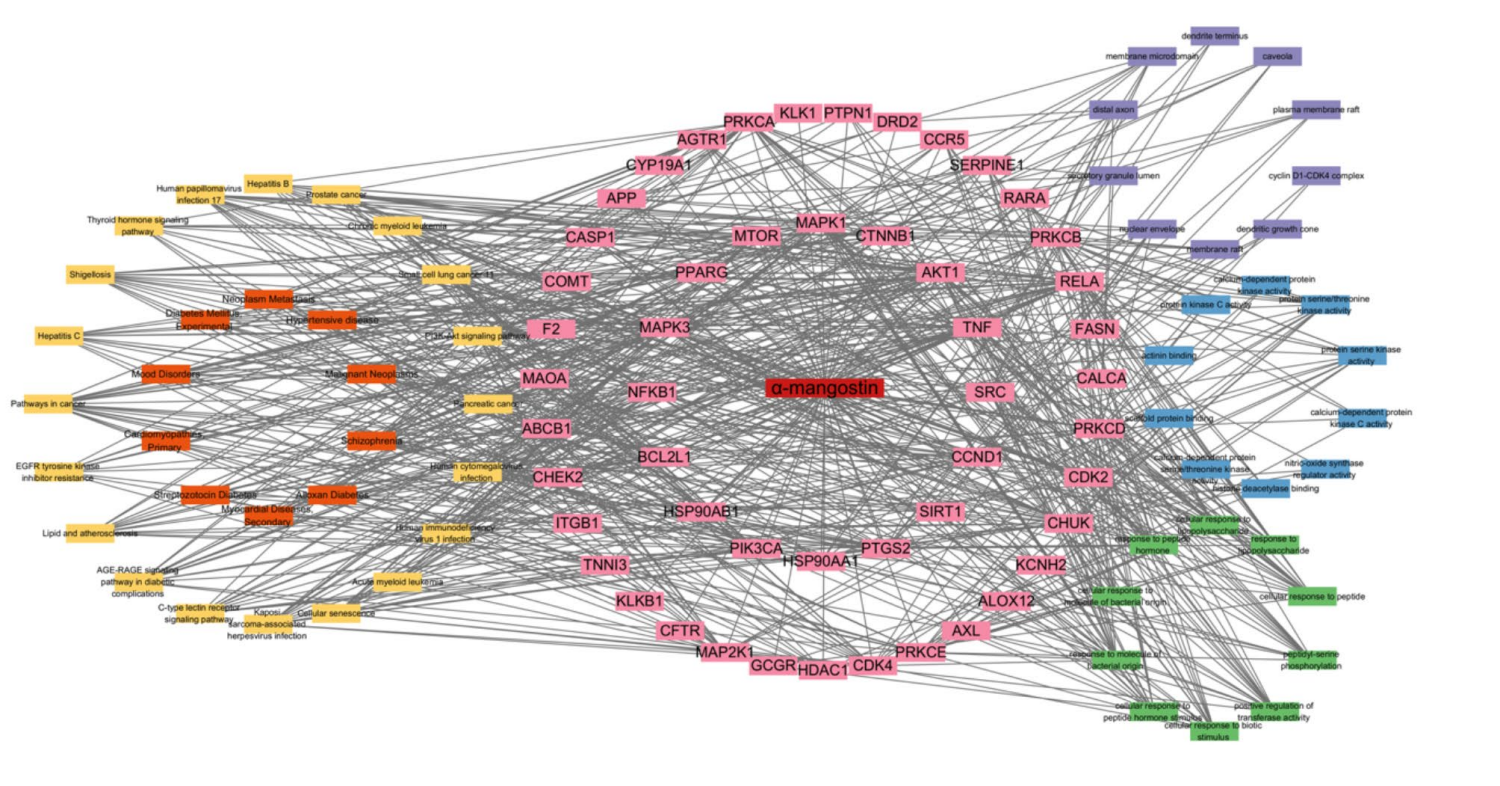
Diseases-targets-functional annotations-signaling pathways network. GO terms are shown on the right side. The purple rectangle represents the cellular component. The blue rectangle represents the molecular function. The green rectangle represents the biological process. The central pink circle represents α-MG targets against hypertension. Diseases enrichment analyses and the enriched KEGG pathways are indicated on the left side. The orange rectangle represents the diseases enrichment analyses. The yellow rectangle represents the enriched KEGG pathways

GO term enrichment, KEGG pathway, and disease enrichment analyses were performed for the target genes shared between alpha-mangostin (α-MG) and hypertension. The top 10 enriched biological processes, molecular functions, and cellular components are depicted in Fig. 3A-3C. Figure 3D highlights the top 20 enriched KEGG pathways, ranked by the number of associated genes. Lastly, the top 10 enriched diseases were selected to construct a disease-gene-function-pathway network, as shown in Fig. 4.

### Assessment of binding capacity between α-MG and therapeutic targets by molecular docking

**Fig. 5:**
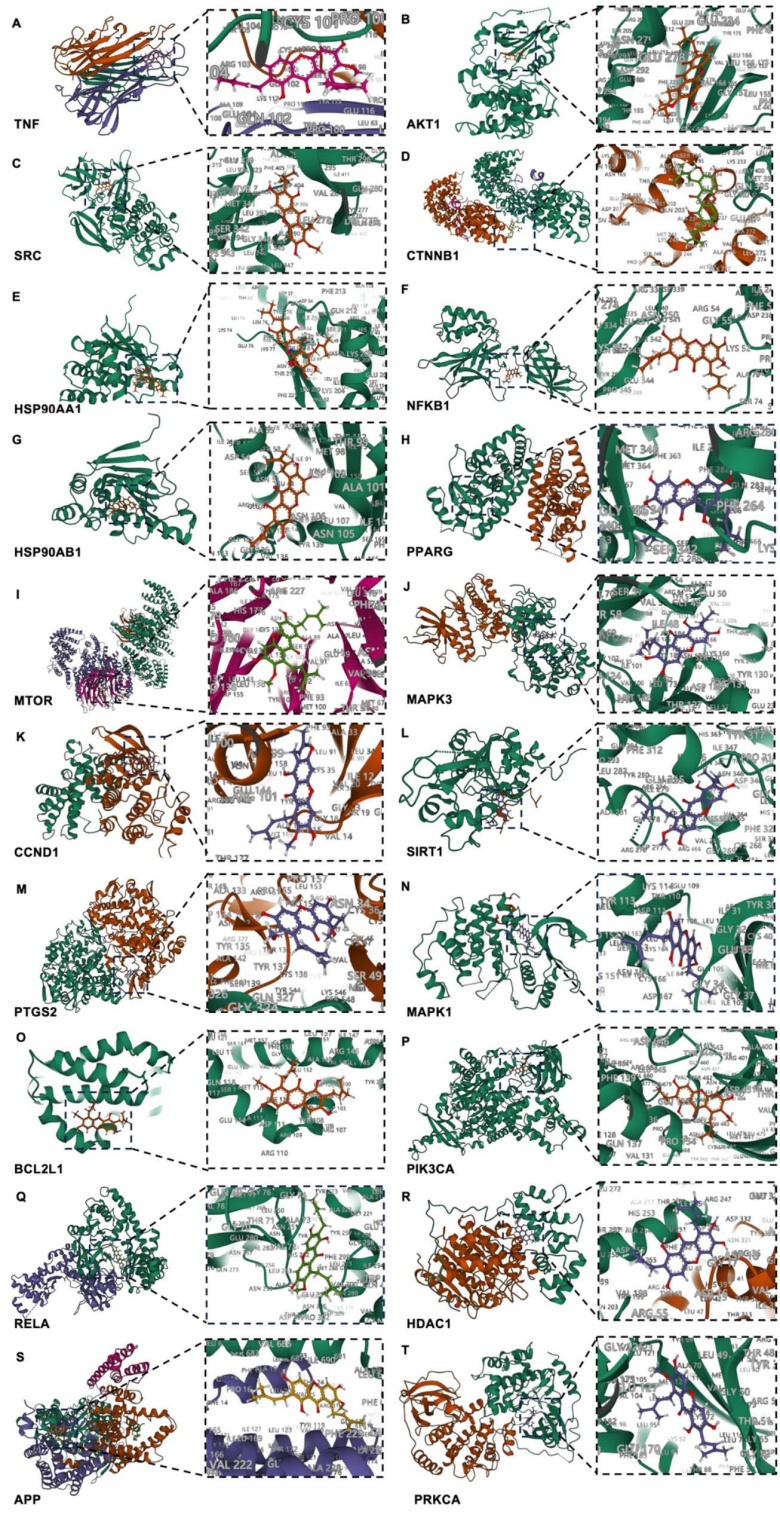
3-D Schematic view of molecular docking of the top 20 core targets of α-MG against hypertension. **A** TNF, **B** AKT1, **C** SRC, **D** CTNNB1, **E** HSP90AA1, **F** NFKB1, **G** HSP90AB1, **H** PPARG, **I** MTOR, **J** MAPK3, **K** CCND1, **L** SIRT1, **M** PTGS2, **N** MAPK1, **O** BCL2L1, **P** PIK3CA, **Q** RELA, **R** HDAC1, **S** APP, **T** PRKCA

To assess the binding affinity of α-MG to the core targets (top 20 genes sorted by DEGREE), we performed molecular docking analysis. We used Autodock Vina v.1.2.2 to obtain the above interactions between α-MG and 20 target proteins. The binding energies of the top 20 core targets with α-MG are all below − 5 kcal/mol, indicating that α-MG has the potential to form stable spatial structures (Tables 2 and 3; Fig. 5). α-MG exhibits the strongest and most stable binding affinity with HSP90AA1.

**Table 3:** Molecular docking of the top 20 core targets of α-MG

Drug	Targets	PDB ID	Binding affinity (kcal/mol)
α-MG	TNF	1TNF	-9.971
α-MG	AKT1	4EKL	-8.478
α-MG	SRC	2SRC	-8.167
α-MG	CTNNB1	1TH1	-6.613
α-MG	HSP90AA1	8B7I	-126.22
α-MG	NFKB1	1SVC	-6.899
α-MG	HSP90AB1	2XJX	-7.732
α-MG	PPARG	6C1I	-8.098
α-MG	MTOR	4JSV	-8.329
α-MG	MAPK3	2ZOQ	-8.813
α-MG	CCND1	2W96	-8.088
α-MG	SIRT1	4KXQ	-8.508
α-MG	PTGS2	5IKQ	-9.099
α-MG	MAPK1	3TEI	-7.567
α-MG	BCL2L1	6GL8	-6.221
α-MG	PIK3CA	6GVH	-7.561
α-MG	RELA	3RC0	-9.414
α-MG	HDAC1	4BKX	-8.384
α-MG	APP	6IYC	-9.396
α-MG	PRKCA	7Y1G	-7.895

### Treatment with α-MG in Ang II-induced hypertensive mice

**Fig. 6:**
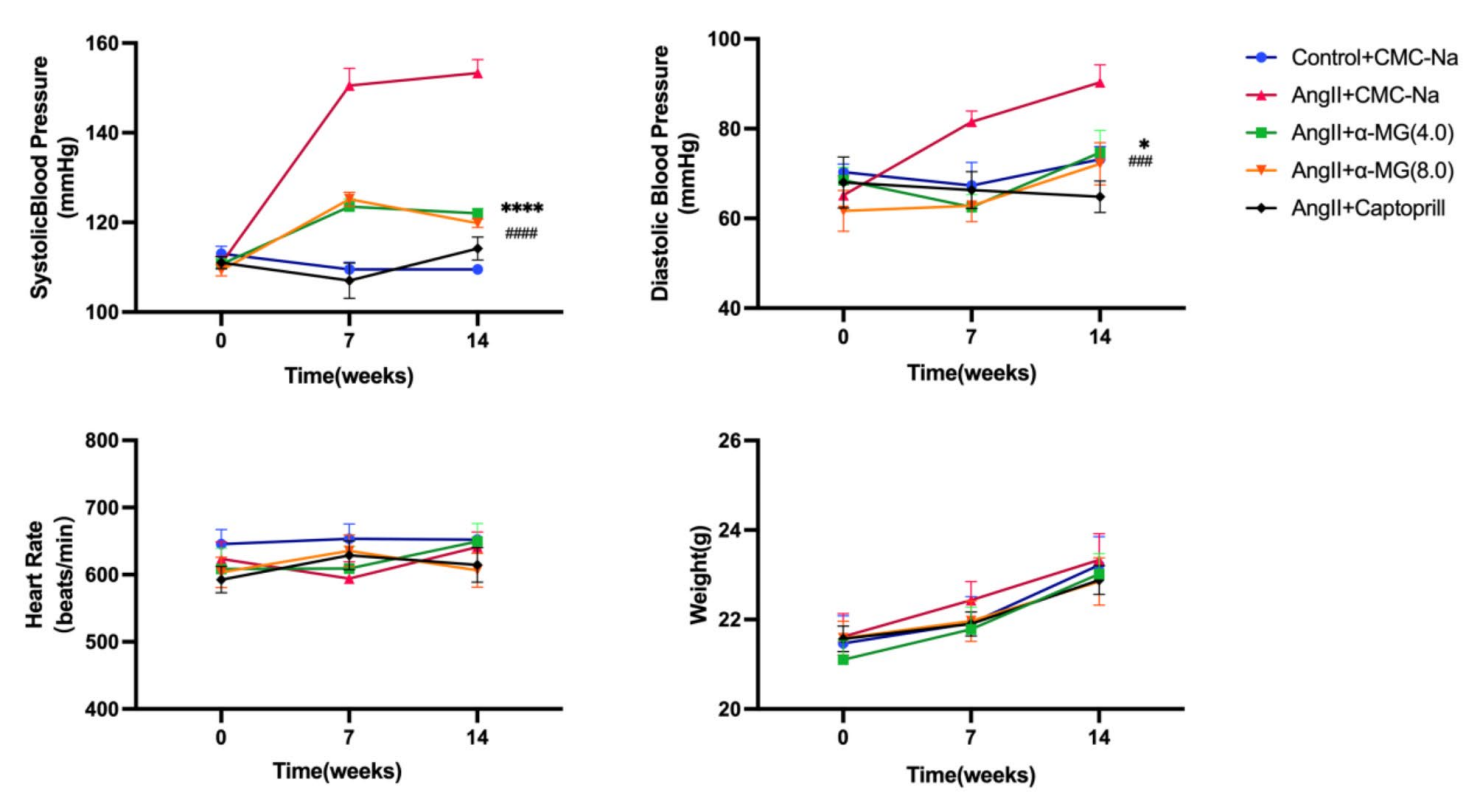
Treatment with α-MG (4.0 mg/kg) reduces blood pressure. A-D. Systolic blood pressure, diastolic blood pressure, heart rate, and weight of control and Ang II-infused hypertensive mice treated with CMC-Na, α-MG (4.0 mg/kg and 8.0 mg/kg) and Captopril twice daily for 14 days. (Values are epresented as mean ± SEM, *n* = 6, ^*^P<0.05, ^****^P<0.0001, ^###^*P* < 0.001 with the two-way ANOVA)

To assess the antihypertensive effect of α-MG, we constructed a mouse model of Ang II infused hypertension. Mice were administered of α-MG by gavage twice daily, and tail-cuff manometry was performed on day 3, 7, and 14. A significant decrease in systolic blood pressure in the mice with α-MG treatment (4.0 mg/kg and 8.0 mg/kg) compared to the Ang II group was observed (Fig. 6). The data indicated that increasing the dose of α-MG did not result in a further reduction in blood pressure. No significant differences in body weight or heart rate were observed between the groups during the experiment.

### Effect of α-MG on target proteins

**Fig. 7:**
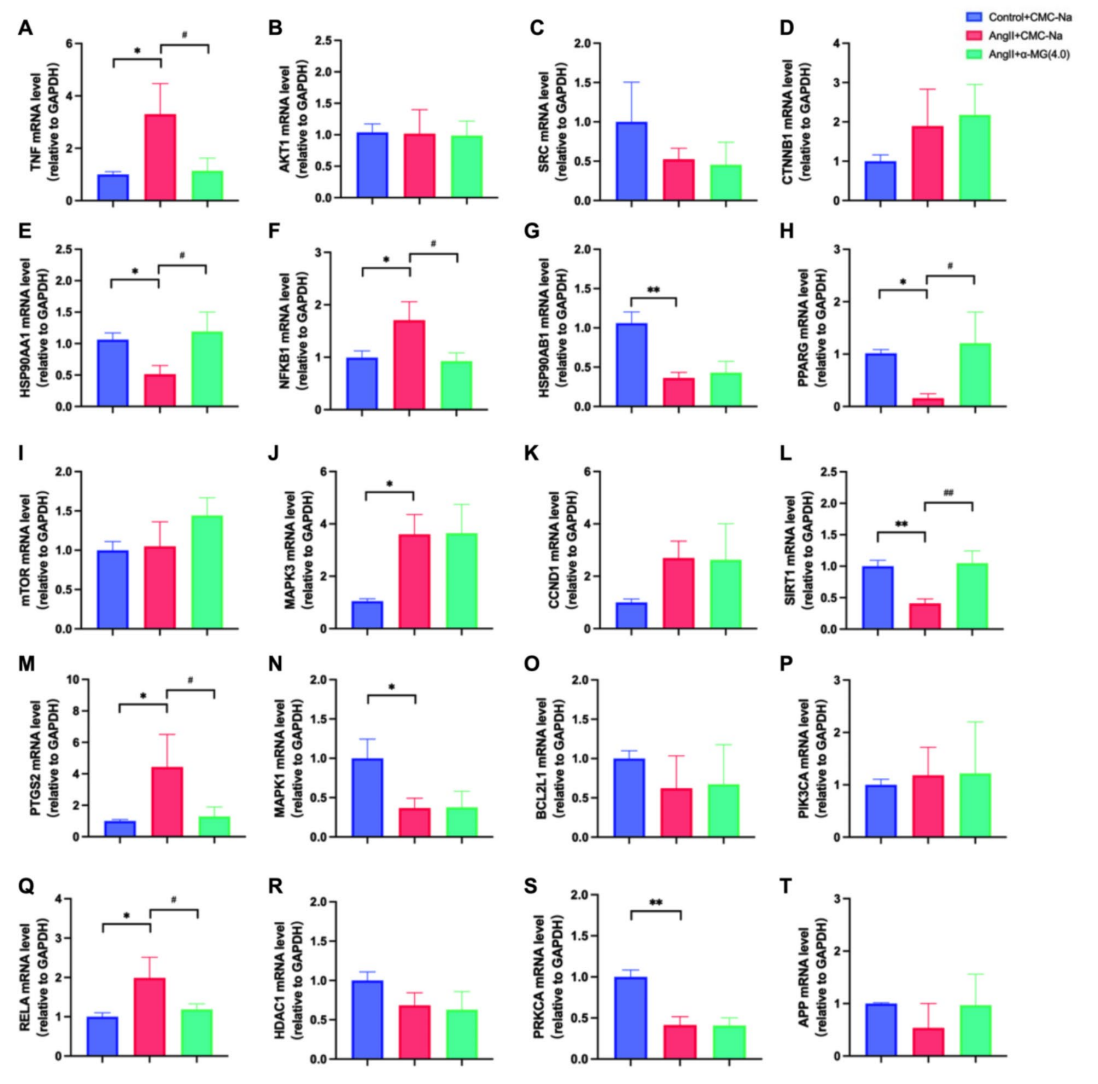
Effect of α-MG on the top 20 targets in mice aorta. The mRNA expression level of **A** TNF, **B** AKT1, **C** SRC, **D** CTNNB1, **E** HSP90AA1, **F** NFKB1, **G** HSP90AB1, **H** PPARG, **I** MTOR, **J** MAPK3, **K** CCND1, **L** SIRT1, **M** PTGS2, **N** MAPK1, **O** BCL2L1, **P** PIK3CA, **Q** RELA, **R** HDAC1, **S** APP, **T** PRKCA in the control, Ang II infusion and α-MG treatment groups are presented as mean ± SD. (*n* = 3. ^*^P<0.05, ^**^P<0.01, ^***^P<0.001)

The mRNA expression levels of the target proteins in mice aorta in the control, Ang II, and α-MG(4.0 mg/kg) treatment groups were examined by RT-qPCR. The results indicate that in the Ang II-induced hypertension group, the expression levels of TNF, NFKB1, MAPK3, PTGS2, and RELA were markedly elevated compared to the control group, while the expression levels of HSP90AA1, HSP90AB1, PPARG, SIRT1, MAPK1, and PRKCA were significantly decreased. Administration of α-MG at a dosage of 4.0 mg/kg twice daily substantially reversed the dysregulated expression of TNF, HSP90AA1, NFKB1, PPARG, SIRT1, PTGS2, and RELA in the Ang II-induced hypertensive mice (Fig. 7).

## Discussion

α-MG, as a notable xanthone present in the fruit of mangostan fruit (Garcinia mangostana Linn.), has garnered attention owing to its various pharmacological properties, including anti-inflammatory, antioxidant, and antimicrobial effects (Juan et al. 2023; Chuang et al. 2021; Yongpitakwattana et al. 2021; Li et al. 2019). Considering hypertension as a chronic disease characterized by inflammatory conditions continues to raise public health concerns, exploring the therapeutic potential of the promising drug α-MG becomes critical. In this study, we for the first time analyzed and identified the mechanisms through which α-MG potentially exerts its antihypertensive properties by integrating network pharmacology, molecular docking and experimental validation.

By constructing a PPI network, we identified 7 core targets of α-MG, including TNF, HSP90AA1, NFKB1, PPARG, SIRT1, PTGS2, and RELA. The satisfying result of molecular docking, as mentioned in the context, indicated highly stable binding of the core targets to α-MG.

The human TNF gene is a 7 kb DNA sequence that encodes TNF-α and TNF-β (Huang et al. 2020). TNF-α is a pleiotropic pro-inflammatory cytokine that plays a crucial role in various diseases, including hypertension (Granger 2006). Heightened concentrations of TNF are associated with augmented vascular resistance and endothelial impairment, both of which are essential elements in the pathophysiology of hypertension (He et al. 2024; Bernier et al. 2024). TNF can trigger the activation of diverse signaling pathways, leading to structural and functional alterations in the vascular system that cause hypertension (Mehaffey and Majid 2017; Kofler et al. 2005). Based on previous studies, α-MG can reduce circulating TNF-α levels in acute liver injury and acute kidney injury (Fu et al. 2018; Eltahir et al. 2023), and our current work further indicated that α-MG may directly target TNF-α in Ang II-induced hypertension.

HSP90AA1 participates in numerous signaling pathways associated with cellular stress responses and inflammation (Condelli et al. 2019; Liu et al. 2021). Although the role of HSP90AA1 has not been implicated in the pathogenesis of hypertension, it has been identified as a hub gene in multiple bioinformatics studies exploring gene expression profiles in pulmonary arterial hypertension (Yang et al. 2024). Several studies have underscored its potential protective role in the pathlogical processes of hypertension. Inhibition of HSP90 was shown to promote vasoconstriction possibily due to reduced bioavailability of NO (Ramírez et al. 2008). HSP90AA1 helps maintain the activity of endothelial nitric oxide synthase (eNOS) (Barrera-Chimal et al. 2014; Luo et al. 2023). As is shown, the docking of α-MG to HSP90AA1 has the lowest binding energy (-126.22 kcal/mol), indicating a strong and stable binding. Therefore, α-MG may exert its antihypertensive effects, at least in part, by interacting with HSP90AA1. The binding affinity of HSP90AA1 is markedly higher compared to other targets. In our docking study, we employed the crystal structure reported in a paper published in December 2022 (Henot et al. 2022), which may introduce some discrepancies with the actual situation. The limitation of molecular docking is that the binding energy only predicts the binding affinity of the drug to the target and cannot verify their actual binding situation, still less the interaction pattern whether the drug affects the target’s activity or expression (Chen 2015; Jiang et al. 2022). Therefore, further experimental validation is indeed necessary.

RELA (p65) and NFKB1 (p50) are crucial members of NFKB family, which plays a crucial role in the inflammatory responses (Hoesel and Schmid 2013; Concetti and Wilson 2018; Ross et al. 2010). p50 and p65 form a dimer and translocate to the nucleus, where they regulate the transcription of pro-inflammatory cytokines such as TNF-α, IL-6, and IL-1β (Zhang et al. 2016; Nakajima and Kitamura 2013). These cytokines are reported to be related with vascular inflammation, endothelial dysfunction, and arterial stiffness (Tuttolomondo et al. 2010). Studies have shown that NFKB activation due to Ang II contributes to inflammation and remodeling in various tissues, notably the heart and kidneys (Hernández-Presa et al. 1997; Zhang et al. 2024; Song et al. 2024). Given Ang II as the key component of the renin-angiotensin system, NFKB activation is critically involved in the development of hypertension (Fu et al. 2024). Consistent with the findings of previous researches that α-MG can suppress the activation of NFKB signaling pathway-related inflammation, we found that a-MG significantly reduced RELA and NFKB1 expression levels in Ang II-infused hypertensive mice (Zou et al. 2019; Pan et al. 2017).

Peroxisome Proliferator-Activated Receptor Gamma (PPARγ) is a member of the nuclear receptor superfamily (Fahmi et al. 2011). Its activation has been shown to improve insulin sensitivity, reduce inflammation, and protect endothelial function (Peters et al. 2008). PPARγ can mitigate vascular dysfunction by enhancing the bioavailability of NO and modulating Ca^2+^ sensitization (Wu et al. 2021; Atkins et al. 2009). It has been shown that PPARγ agonists exhibit a notable antihypertensive effect in spontaneously hypertensive rats (Wakino et al. 2005; Lewington et al. 2002). Considering its substantial role in the pathophysiology of hypertension, PPAR-γ may be one of the crucial anti-hypertensive targets of α-MG.

SIRT1 is an NAD^+^-dependent deacetylase that targets both histone and non-histone proteins for deacetylation (Yang et al. 2022). It has been reported that activation of SIRT1 suppressed AT1R expression and enhanced NO bioavailability, thereby ameliorating hypertension in mice (Miyazaki et al. 2008; Cui et al. 2012). In the hypertensive paraventricular nucleus (PVN) of the hypothalamus, inhibition of SIRT1 has been shown to promote inflammasome activation, aggravating hypertension (Jia et al. 2023). Wu et al. showed that α-MG upregulated SIRT1 in macrophages and inhibited inflammation (Chen et al. 2022). In line with our study, we observed decreased SIRT1 expression in Ang II-infused hypertensive mice, and treatment with α-MG reversed abnormal SIRT1 expression, suggesting that SIRT1 may also be an important target of α-MG, although detailed mechanisms remain to be further elucidated.

PTGS2, or cyclooxygenase-2 (COX-2), is a pivotal enzyme converting arachidonic acid to prostaglandins, and is elevated under inflammatory status (Haase-Kohn et al. 2022). PTGS2 is reported to exacerbate vascular inflammation and endothelial dysfunction by enhancing reactive oxygen species (ROS) and inflammatory response (Martínez-Revelles et al. 2013). Dysregulation of PTGS2 has been shown to lead to sodium and water retention (Chen et al. 2008). Previous studies have demonstrated that Ang II can induce PTGS2 expression and prostaglandin production in various vascular cell types (Martínez-Revelles et al. 2013). Our findings also showed elevated PTGS2 expression in the aorta of Ang II-infused hypertensive mice, which was reversed by treatment with α-MG, indicating that PTGS2 not only served as a critical player in molecular mechanism of hypertension but also as a therapeutic target of α-MG.

The enrichment analysis of Gene Ontology and KEGG pathway may provide insights into the underlying mechanisms of hypertension and therapeutic targets. The present study showed that pathways in cancer, human cytomegalovirus infection, prostate cancer and chronic myeloid leukemia, and AGE-RAGE signaling pathway in diabetic complications might be involved in the effect of key therapeutic targets of α-MG. Given the current significant amount of research of α-MG in cancer therapy, it is understandable that the pathways enriched utilizing the existing databases are primarily pathways associated with cancer. However, it still offers insights into the possible pathways and biological processes in relation to hypertension and α-MG, such as chronic inflammation, oxidative stress, and metabolic disturbances (Wilcox et al. 2024; Tiwari and Dwivedi 2019; Berg et al. 2023).

To date, no studies have comprehensively screened and validated the targets of α-MG in the context of hypertension treatment. Our findings provide a theoretical foundation for the pharmacological research of α-MG in ameliorating hypertension and associated target organ damage. However, this study has also some limitations. Firstly, network pharmacology and molecular docking are highly reliant on the quality of databases and the accuracy of algorithms. Due to the inherent limitations of these databases and software, our understanding of drug mechanisms and potential interactions remains incomplete. Besides, taking hub nodes as targets imply these genes with high degree value play crucial role in the pathogenesis of hypertension. However, some important genes may be ignored. Secondly, the identified targets were validated only through computational simulations and in vivo experiments, without the construction of multiple hypertension models for comprehensive in vivo validation. Additional experiments with larger sample size to elucidate the detailed molecular mechanisms by which α-MG ameliorates hypertension and target organ damage are warranted. Thirdly, time and resource constraints prevented us from experimentally confirming all predicted targets or integrating animal experiments with clinical validation. Finally, the current findings do not offer guidance on the optimal intervention dose of α-MG for clinical applications. Therefore, further pharmacological studies are needed.

## Electronic supplementary material

Below is the link to the electronic supplementary material.

Supplementary Material 1

## Data Availability

No datasets were generated or analysed during the current study.
