# Supplementary material for: Decoding the anti-hypertensive mechanism of α-mangostin based on network pharmacology, molecular docking and experimental validation
Source: Mol Med. 2024 Nov 26;30:234. doi: 10.1186/s10020-024-01001-8 (PMC11600633; doi:10.1186/s10020-024-01001-8)
Supplement: Supplementary file 1 — Supplementary Material 1 [file 10020_2024_1001_MOESM1_ESM.docx]

**Decoding the Anti-hypertensive Mechanism of α-Mangostin Based on Network Pharmacology, Molecular Docking and Experimental Validation**

Qi-Qi Xue*, Chu-Hao Liu*, Yan Li

^1^ Department of Cardiovascular Medicine, Shanghai Institute of Hypertension, Shanghai Key Laboratory of Hypertension, National Research Centre for Translational Medicine, Ruijin Hospital, Shanghai Jiaotong University School of Medicine, Shanghai, China

*Co-first Author

Correspondence to: Dr. Yan Li, Department of Cardiovascular Medicine, Shanghai Institute of Hypertension, Ruijin Hospital, Shanghai Jiaotong University School of Medicine, Ruijin 2nd Rd 197, Shanghai 200025, China, E-Mail: [liyanshcn@163.com](mailto:liyanshcn@163.com).

| Gene Name | A | B | C | Multiple Comparisons | |
| --- | --- | --- | --- | --- | --- |
|  | Control | AngII+CMC-Na | AngII+α-MG (4.0) | *P-value* (A vs. B) | *P-value* (B vs. C) |
| TNF | 1.01±0.09 | 3.30±0.95 | 1.14±0.39 | 0.017 | 0.022 |
| AKT1 | 1.04±0.11 | 1.02±0.31 | 0.99±0.19 | 0.990 | 0.995 |
| SRC | 1.16±0.60 | 0.51±0.19 | 0.56±0.31 | 0.966 | 0.262 |
| CTNNB1 | 1.07±0.16 | 2.26±0.98 | 2.59±0.45 | 0.873 | 0.316 |
| HSP90AA1 | 1.01±0.07 | 0.52±0.19 | 1.01±0.02 | 0.013 | 0.033 |
| NFKB1 | 1.03±0.15 | 1.85±0.35 | 0.84±0.06 | 0.013 | 0.020 |
| HSP90AB1 | 1.00±0.13 | 0.39±0.08 | 0.35±0.05 | 0.770 | 0.001 |
| PPARG | 0.99±0.08 | 0.20±0.05 | 1.14±0.82 | 0.020 | 0.046 |
| MTOR | 0.97±0.13 | 0.87±0.06 | 1.48±0.30 | 0.963 | 0.158 |
| MAPK3 | 1.09±0.09 | 3.27±0.68 | 4.03±1.23 | 0.997 | 0.013 |
| CCND1 | 1.05±0.13 | 2.85±0.84 | 2.07±1.37 | 0.996 | 0.109 |
| SIRT1 | 1.00±0.09 | 0.41±0.07 | 1.05±0.19 | 0.002 | 0.003 |
| PTGS2 | 1.00±0.09 | 4.44±2.06 | 1.29±0.60 | 0.042 | 0.029 |
| MAPK1 | 1.00±0.24 | 0.37±0.12 | 0.38±0.20 | 0.998 | 0.015 |
| BCL2L1 | 1.00±0.10 | 0.62±0.41 | 0.67±0.50 | 0.464 | 0.985 |
| PIK3CA | 1.00±0.11 | 1.18±0.53 | 1.22±0.98 | 0.997 | 0.934 |
| RELA | 1.00±0.1 | 1.99±0.52 | 1.19±0.14 | 0.043 | 0.018 |
| HDAC1 | 1.00±0.19 | 0.68±0.28 | 0.63±0.40 | 0.433 | 0.971 |
| APP | 1.00±0.02 | 0.54±0.46 | 0.97±0.59 | 0.461 | 0.420 |
| PRKCA | 1.02±0.20 | 0.34±0.17 | 0.41±0.23 | 1.000 | 0.009 |

Table S1: Statistical data for mRNA expression levels
